# Life’s Mirror

**Published:** 1916-10

**Authors:** Madeline S. Bridges


### EDITORIAL DEPARTMENT

MRS. F. E. DANIEL, Publisher and Managing Editor.

### Life's Mirror.

There are loyal hearts, there are spirits brave,
There are souls that are pure and true;
Then give to the world the best you have,
And the best will come back to you.

Give love, and love to your heart will flow,
A strength in your utmost need;
Have faith and a score of hearts will show
Their faith in your word and deed.

For life is the mirror of kind and slave,
'Tis just what you are and do;
Then give to the world the best you have,
And the best will come back to you.

—*Madeline S. Bridges.*

